# Investigating the effect of hippocampal sclerosis on parietal memory network

**DOI:** 10.1002/epi4.12870

**Published:** 2023-12-21

**Authors:** Silke Ethofer, Monika Milian, Michael Erb, Sabine Rona, Jürgen Honegger, Thomas Ethofer

**Affiliations:** ^1^ Department of Neurosurgery University Hospital Tübingen Tübingen Germany; ^2^ Department of Biomedical Magnetic Resonance University of Tübingen Tübingen Germany; ^3^ Department of Psychiatry and Psychotherapy University Hospital Tübingen Tübingen Germany; ^4^ Present address: Klinik Lengg AG, Swiss Epilepsy Clinic Zurich Switzerland

**Keywords:** episodic memory, fMRI, nonverbal memory, parietal memory network, precuneus, temporal lobe epilepsy

## Abstract

**Objective:**

We aimed to investigate differences in episodic memory networks between patients with temporal lobe epilepsy (TLE) due to hippocampal sclerosis and healthy controls, especially with regards to the parietal memory network (PMN), as well as their relation to neuropsychological memory performance after mesial temporal resection.

**Methods:**

28 healthy subjects as well as 21 patients with TLE (12 left, 9 right) were investigated using a spatial memory fMRI paradigm, which has been shown to activate the PMN. Regions of interest (ROI) were defined based on the results of the second‐level analyses and activations within the predefined ROIs were compared across groups and correlated with postoperative verbal and nonverbal memory scores.

**Results:**

Healthy subjects showed activations within regions belonging to the dorsal visual stream and the PMN as well as the bilateral parahippocampal place area, the bilateral frontal eye field, and the bilateral middle frontal gyrus. Comparison between groups revealed that TLE patients activated significantly less in the left middle occipital gyrus and the right precuneus. The activation pattern in left TLE patients showed further reductions, mainly in areas belonging to the dorsal visual stream and the PMN within the left hemisphere. Activations within the left superior parietal lobulus, bilateral inferior parietal lobulus, bilateral middle temporal gyrus, left precuneus, left frontal eye field, and left middle frontal gyrus correlated significantly with postoperative verbal memory scores, and activations within the left superior parietal lobulus, left inferior parietal lobulus, left middle temporal gyrus, and left precuneus correlated significantly with higher performance in postoperative nonverbal memory scores.

**Significance:**

The PMN is involved in episodic memory encoding. Higher activations in areas belonging to the PMN and the dorsal visual stream, especially within the left hemisphere, before amygdalohippocampectomy may result in higher postoperative memory scores.

**Plain language summary:**

This study aims to investigate the effects of epilepsy due to hippocampal sclerosis, i.e. scarring in the temporal lobe, on memory networks in the brain. We discovered that especially patients with left‐sided hippocampal sclerosis show reduced brain activations in visual areas and memory networks within the left hemisphere of the brain during orientation in space. Importantly, higher activations within these areas may result in better memory after epilepsy surgery.

**Key points**

- Left TLE patients show decreased activations in the left dorsal visual stream and parietal memory network (PMN).
- Activity in the left precuneus, superior occipital gyrus, and middle temporal gyrus correlates with postoperative verbal and nonverbal memory scores and activity in the left superior and inferior parietal lobulus, the left superior frontal gyrus, and the bilateral middle occipital and middle frontal gyrus correlates with postoperative verbal memory scores.
- Higher activations in the PMN and the dorsal visual stream before amygdalohippocampectomy may result in higher postoperative memory scores.

## 1 INTRODUCTION

The mesial temporal lobe (mTL), especially the hippocampus (HC), is considered to play a pivotal role in declarative memory. It is supposed to be a fast learner and to act as temporary storage for new information, while memory consolidation and permanent storage depends on a broadly distributed cortical network.^1^, ^2^ However, in recent years the so‐called parietal memory network (PMN) consisting of precuneus, mid‐cingulate cortex, and posterior inferior parietal lobule, and its role in learning and memory has gained more and more scientific attention.^3^ It has been shown to be more active with increasing repetition or familiarity of a stimulus,^3^ while hippocampal activity is strongest during early encoding and diminishes over time.^2^ However, the exact relationship between HC and PMN is a debate of ongoing research. Resting‐state fMRI studies have demonstrated robust correlations between HC and PMN.^4^ Connectivity between HC and PMN is strongest during initial encoding and declines with increasing repetition.^2^

But what happens to the PMN if hippocampal function is impaired? Investigating memory function in patients with hippocampal lesions can open up new perspectives on the interplay between HC and PMN. It offers the possibility to find out in what way the PMN is affected by hippocampal pathology and involved in possible reorganization processes. While it is common knowledge that the dominant HC predominates in mediating verbal memory functions^5^ and the nondominant in nonverbal memory functions,^6^ little is known about the lateralization of the PMN or the dependence on the HC of the dominant versus the nondominant hemisphere.

Hippocampal sclerosis (HS) is a common cause of pharmacoresistant temporal lobe epilepsy (TLE) and selective resection has been validated as an effective therapy in these cases as it eliminates seizures or improves seizure control in 50–80% of cases.^7^, ^8^ However, surgery within the mTL bears the risk of significant decline in episodic memory function.^9^ The understanding of memory processes, especially with regards to memory outcome after mTL resection is, therefore, of particular importance in this patient cohort.

The aim of the current study was the investigation of differences in episodic memory networks between patients with TLE due to HS and healthy controls using fMRI. We applied a spatial memory paradigm, where subjects had to encode and recognize the location of geometrical objects in a virtual three‐dimensional environment, which has been shown to activate the PMN.^10^, ^11^ We hypothesized that patients with TLE due to HS differ from healthy controls regarding activations within the PMN. Furthermore, we addressed whether these activations can predict memory performance before and after mTL resection.

## 2 MATERIALS AND METHODS

### 2.1 Participants

Twenty‐one right‐handed TLE patients with unilateral HS, who underwent presurgical evaluation at the University Hospital Tübingen, were included in the study. All patients had clear signs of unilateral HS on structural MRI as determined by experienced neuroradiologists, including unilateral hippocampal atrophy and increased T2 signal intensity. Twelve patients had left‐sided HS (7 females, mean age 36.6 years, SEM 3.6, range 18–57), in the following referred to as LTLE patients, and nine had right‐sided HS (2 females, mean age 52.2 years, SEM 4.6, range 21–70), to which we will refer as RTLE patients. There were no patients with dual lesions. Twelve of these patients underwent mTL resection (7 left). For better understanding, these patients will be referred to as LTLE‐post and RTLE‐post in the following. For further details regarding patients' characteristics see Table 1.

**TABLE 1:** Demographic data of patients (*N* = 21).

Patients	Age/sex	Age at epilepsy onset (years)	Duration of epilepsy (years)	Seizure frequency (seizures/month)	mTL resection performed yes/no	ASM
Left HS group						
1	30/F	15	15	8	No	LEV, LTG
2	36/M	14	22	6	No	LCM, LEV
3	47/M	19	28	2	Yes	LEV, RTG
4	57/F	22	35	20	Yes	LEV
5	46/M	37	9	20	Yes	LEV, OXC, RTG
6	49/F	1	48	20	Yes	CBZ, CZP, LEV
7	36/F	12	24	5	Yes	LCM, LEV, LTG, TPM
8	46/M	17	29	12	Yes	LCM, LTG
9	25/F	15	10	5	Yes	LTG
10	28/F	19	9	4	Yes	LCM, LEV, LTG
11	21/M	11	10	2	No	OXC
12	18/F	13	5	0.25	No	VPA
Mean (SEM)	36.6 (3.6)	16.3 (2.4)	20.3 (3.8)	10.8 (2.4)		
Right HS group						
1	53/M	0	53	20	Yes	CBZ, LEV
2	70/F	5	65	12	No	LCM, LEV
3	51/M	48	3	0.33	Yes	ESL, LEV
4	64/M	4	60	1	No	LTG, VPA, ZNS
5	57/F	45	12	13	No	LCM, LEV
6	51/M	10	41	30	No	CBZ, LCM, PB, RTG
7	21/M	5	16	28	Yes	VPA
8	57/M	43	14	3	Yes	LTG
9	46/M	3	43	1	Yes	LCM, PER
Mean (SEM)	52.2 (4.6)	18.1 (6.9)	34.1 (7.7)	15.7 (4.2)		

Abbreviations: ASM, antiseizure medication; CBZ, carbamazepine; CZP, clonazepam; ESL, eslicarbazepine acetate; F, female; HS, hippocampal sclerosis; LCM, lacosamide; LEV, levetiracetam; LTG, lamotrigine; M, male; mTL, mesial temporal lobe; OXC, oxcarbazepine; PB, phenobarbital; PER, perampanel; RTG, retigabine; SEM, standard error of the mean; TPM, topiramate; VPA, valproate; ZNS, zonisamide.

The cohort of healthy controls consisted of 28 right‐handed participants (21 female, mean age 28.0 years, SEM 1.2, range 18–46). All participants were native speakers of German and strongly right‐handed as assessed by the Edinburgh Inventory (mean handedness quotient >0.97).^12^ The study was approved by the Ethics committee of the University of Tübingen and was in accordance with the guidelines of the Declaration of Helsinki. All participants gave written informed consent.

### 2.2 Neuropsychological tests

Verbal learning and memory were evaluated using a wordlist learning and retention test which required memorizing a list of 15 words (Verbaler Lern‐ und Merkfähigkeitstest, VLMT).^13^, ^14^ To assess nonverbal learning and memory, we used the revised version of the DCS (Diagnostikum für Cerebralschädigung),^15^ during which subjects had to memorize nine geometrical figures. In both tests, we assessed the “immediate recall” memory score, that is, the sum of correctly reproduced items during five learning trials. It is known that memory performance levels decrease with age.^16^, ^17^ Therefore, we used the percentile ranks of both memory tests instead of the raw scores, that is, the standardized memory performance as compared to an age‐matched reference population, for the analyses. The lowest percentile rank in the VLMT according to the manual is PR <5, that is, a range between 0 and 4.9.^18^ For calculation purposes, we used PR = 0 for these cases. As recalculation with PR = 4.9 instead of PR = 0 gave identical results up to the third position after the decimal point, the use of PR = 0 seemed justified.

In the patients who underwent epilepsy surgery (LTLE‐post and RTLE‐post), memory scores were assessed before and at least 12 months after epilepsy surgery to guarantee postoperative memory consolidation (mean 17.2 months, SEM 1.8). The level of verbal crystallized intelligence was determined using the German multiple choice vocabulary test (MWT‐B, Mehrfachwahl‐Wortschatz‐Intelligenztest),^19^, ^20^ which has been shown to correlate with the Full Scale IQ of the HAWIE‐R.^21^

### 2.3 Acquisition of MRI data

MRI data were acquired using a Siemens Magnetom Sonata [Maestro Class] 1.5 T Scanner (Siemens AG) with an 8‐channel array head coil for reception and the body coil for transmission. A sagittal T1‐weighted 3D‐MPRAGE sequence was used to obtain a high‐resolution anatomical image of each subject's brain (TR/TI/TE = 1300/660/3.19 ms, flip angle 15°, field‐of‐view = 256 × 256 mm^2^, matrix 256 × 256, 176 slices, voxel size 1 × 1 × 1 mm^3^). Additionally, a field map was recorded for distortion correction of the functional images caused by magnetic field inhomogeneity. For the MRI task, 175 gradient‐echo planar T2*‐weighted images covering the whole brain were acquired (TR = 4000 ms, TE = 64 ms, field‐of‐view 192 × 192 mm^2^, matrix 64 × 64, voxel size 3 × 3 × 3 mm^3^, gap = 0.3 mm, 38 interleaved slices). The first two images of each experimental run were discarded in order to reach equilibrium of magnetization. These parameters were chosen based on a previous study, in which we demonstrated that for functional activations within the hippocampus, acquisition at a TE = 64 ms results in a higher sensitivity of BOLD signal detection within the hippocampus than at a TE = 45 ms.^22^ To achieve this long TE and a full brain coverage with 38 slices a TR = 4 seconds was necessary.

### 2.4 Stimuli and fMRI task design

We used a paradigm that was designed to investigate spatial memory functions by encoding and immediate recognition of object locations within a virtual 3D‐environment and was already described previously.^10^, ^23^ The paradigm comprised four encoding blocks, each consisting of a short video sequence (28 seconds), during which subjects were presented a virtual flight through a labyrinth passing five differently colored geometric objects and were asked to remember their location. Each encoding block was followed by a control condition during which subjects were presented two differently sized versions of the five objects and were asked to indicate by button press on which side the larger one was (duration 24 seconds, 8 items/block). To ensure the participants' attention and compliance during the encoding condition, a recognition task was performed inside the scanner. It was designed as a two‐alternative forced choice test, in which aerial views of the labyrinth were presented containing one of the objects, either at the correct or at a new location in a balanced pseudorandomized order (duration 28 seconds, 5 items/block). Subjects were asked to indicate by button press, whether the object was at the correct location. The recognition task also included four activation blocks alternating with four blocks of the control condition.

The stimuli were presented using Presentation software (Neurbehavioral Systems Inc., http://www.neurobs.com) and were projected on a translucent screen positioned at the end of the scanner table via a video projector outside the magnet and subjects saw them through a mirror attached to the head coil. They conveyed their responses via use of a two‐button box with their right thumb.

### 2.5 Statistical analysis of fMRI data

fMRI data were analyzed in MATLAB R2018b (http://www.mathworks.com) using Statistical Parametric Mapping (SPM12, Wellcome Trust Centre for Imaging Neuroscience; http://fil.ion.ucl.ac.uk/spm). Each subject's imaging time series was corrected for difference in slice acquisition time, realigned and unwarped based on the estimated field map data,^24^ coregistered to the anatomical reference image, normalized to MNI space (Montreal Neurological Institute Atlas),^25^ then smoothed with an isotropic Gaussian kernel (8 mm full‐with at half maximum) and filtered with a high‐pass filter with a cutoff time of 128 seconds.

For first‐level analyses, experimental task and control blocks were defined by a box‐car function of 28 seconds (experimental task) and 24 seconds (control blocks) duration and convolved with the hemodynamic response function. Realignment parameters were added as regressors of no interest. Contrast images of individual main effects for the encoding versus control condition were calculated and second‐level analyses using one‐sample *t*‐tests were performed in order to examine task‐related group main effects. Results are reported at a height threshold of *P* < 0.001 (uncorrected). Correction for multiple comparisons (*P* < 0.05, corrected) across the whole brain was assessed at cluster level using random field theory and only clusters exceeding an extent threshold of *k* ≥ 44 voxels, 45 voxels, and 57 voxels for the LTLE, RTLE and healthy controls group, respectively, were considered for further analysis. Regions of interest (ROI) were defined based on the second‐level results of each group, that is, healthy controls, LTLE and RTLE patients, and masks were created using the automated anatomical labelling atlas (AAL).^26^ These masks were then applied to each subject's first‐level results to extract activations, that is, *beta* estimates, from each ROI to use for further analyses.

Furthermore, second‐level analyses using two‐sample *t*‐tests were performed to compare activations between groups. The results of the two‐sample *t*‐tests are reported at a height threshold of *P* < 0.001 (uncorrected). Correction for multiple comparisons (*P* < 0.05, corrected) across the whole brain was assessed at cluster level using random field theory. The extent threshold for each contrast is given in the results section.

### 2.6 Statistical analysis of behavioral data and correlation with fMRI data

Data were analyzed using IBM SPSS Statistics version 28 (http://www.spss.com). Descriptive statistics were used to analyze sociodemographic and neuropsychological characteristics using minimum, maximum, mean, and standard error of the mean (SEM). We calculated the percentages of correct answers for the fMRI task. Differences between groups in behavioral performances were compared with a one‐way analysis of variance (ANOVA) with behavioral data as dependent variable and group as independent variable. Significant effects in the ANOVA were characterized further using planned contrasts. The first contrast was defined to compare the cohort of patients (LTLE + RTLE) with the healthy controls, the second to compare the two patient groups (LTLE vs. RTLE). To compare differences between both patient groups regarding the demographic factors, two‐sample *t*‐tests were used.

To remove variance correlated with participants' age from the extracted fMRI activations from each ROI in each participant, we performed a simple regression analysis with age as independent variable and event‐related responses in each ROI as dependent variables. Only the regression residuals were used for further analyses. Activations within the ROIs were compared between groups using one‐way ANOVAs as described above. Furthermore, activations were correlated with postoperative verbal and nonverbal memory scores. Correlation analyses were performed across all groups due to the small sample size of operated patients (LTLE‐post + RTLE‐post). To test for normal distribution, Kolmogorov–Smirnov tests were used with *P* > 0.05 indicating normal distribution. To account for the fact that percentile ranks are ranked data, we used Spearman's Correlations.

We report the results of comparisons and correlations descriptively at an uncorrected threshold of *P* < 0.05 to enable future meta‐analyses. However, we additionally employed a correction for multiple comparisons based on the Bonferroni correction. Regarding comparisons of activations in 18 ROIS the adjusted level of significance is *P* < 0.003 (i.e., 0.05/18). With respect to the correlation analyses the adjusted level of significance is *P* < 0.0014 (i.e., 0.05/36) as we calculated 36 correlations.

To determine whether age at epilepsy onset, duration of epilepsy, or seizure frequency are associated with the activations within our ROIs, correlation analyses were performed between these variables and our ROIs. The significance level was set at *P* < 0.05.

## 3 RESULTS

### 3.1 Neuropsychological performance

Comparison of demographic factors between both patient groups revealed only significant differences regarding participants' age (*t* (19) = −2.727, *P* = 0.013), all other *P* > 0.05. The results of the neuropsychological tests are presented in Table 2. ANOVAs indicated that the three groups differed statistically significant regarding IQ (*F*_(2, 46)_ = 13.93, *P* < 0.001, *ω* ^2^ = 0.35), VLMT (*F*_(2, 46)_ = 19.65, *P* < 0.001, *ω* ^2^ = 0.43), and DCS (*F*_(2, 46)_ = 8.59, *P* < 0.001, *ω* ^2^ = 0.24). Planned contrasts revealed that patients (LTLE and RTLE) performed significantly worse than healthy controls in all tests (IQ: *t* (46) = −4.89, *P* < 0.001; VLMT: *t* (46) = −5.99, *P* < 0.001; DCS: *t* (46) = −4.12, *P* < 0.001), while there was no statistically significant difference between both patient groups (LTLE vs. RTLE) (IQ: *t* (46) = −1.45, *P* = 0.153; VLMT: *t* (46) = −1.19, *P* = 0.239; DCS: *t* (46) = 0.002, *P* = 0.999). Interestingly, a correlation analysis between VLMT and DCS scores in TLE patients revealed a significant correlation (*r* = 0.610, *P* = 0.003). However, this observation could not be made in the group of healthy controls (*r* = 0.135, *P* = 0.492). The reason for this most probably lies in the verbalizability of visual material. It has been shown that verbalization can both improve and hinder visual memory performance. RTLE patients are thought to rely on “left hemispheric,” that is, verbal, capacities for the encoding of visual material in order to improve performance. LTLE patients on the other hand might perform worse in nonverbal memory tests due to poorly encoded verbalizations as a result of their verbal memory deficits.^27^, ^28^ The DCS is a nonverbal memory test in which items can be named, but the information content exceeds verbal memory capacity in order to reduce this major confounding factor.^27^ But apparently in patients with memory deficits, either verbal or nonverbal, this factor still comes into play.

**TABLE 2:** IQ and memory performance in healthy subjects, LTLE and RTLE patients.

Group and variables	Minimum	Maximum	Mean (SEM)
Healthy subjects (*n* = 28)			
IQ (MWT‐B)	90.0	145.0	121.3 (3.2)
VLMT PR	5.0	100.0	75.0 (5.1)
DCS PR	12.3	100.0	71.2 (4.1)
LTLE (*n* = 12)			
IQ (MWT‐B)	79.0	118.0	94.1 (3.2)
VLMT PR	0.0	85.0	20.0 (8.3)
DCS PR	5.0	92.0	37.2 (10.0)
LTLE operated (*n* = 7)			
VLMT PR preop	0.0	30.0	10.0 (4.9)
VLMT PR postop	0.0	20.0	5.7 (3.2)
DCS PR preop	5.0	70.8	25.3 (9.7)
DCS PR postop	0.0	47.0	18.5 (6.9)
RTLE (*n* = 9)			
IQ (MWT‐B)	89.0	136.0	104.1 (5.7)
VLMT PR	0.0	90.0	34.4 (8.7)
DCS PR	0.0	81.5	37.1 (12.4)
RTLE operated (*n* = 5)			
VLMT PR preop	0.0	90.0	39.0 (15.0)
VLMT PR postop	20.0	95.0	54.0 (12.6)
DCS PR preop	5.0	81.5	52.3 (16.1)
DCS PR postop	8.6	70.8	41.0 (9.8)

Abbreviations: DCS, Diagnostikum für Cerebralschädigung; LTLE, left temporal lobe epilepsy; MWT‐B, Mehrfachwahl‐Wortschatz‐Intelligenztest (German multiple choice vocabulary test); PR, percentile ranks; RTLE, right temporal lobe epilepsy; VLMT, Verbaler Lern‐ und Merkfähigkeitstest (wordlist learning and memory test).

Comparison of pre‐ and postoperative memory performances using paired samples *t*‐tests (LTLE vs LTLE‐post and RTLE vs. RTLE‐post) only revealed a tendency toward improvement in VLMT scores in the RTLE group (*t* (4) = −2.739, *p* = 0.052). All other comparisons showed no statistically significant differences, especially no significant postoperative memory loss (all *P* > 0.05).

Analysis of fMRI behavioral data revealed 55.0 ± 5.8 percent correctly recognized object locations in the LTLE group, 43.3 ± 8.4 in the RTLE group and 75.0 ± 3.7 in the group of healthy controls. The one‐way ANOVA revealed that the three groups differed statistically significant (*F*_(2, 42)_ = 8.67, *P* < 0.001, *ω* ^2^ = 0.25) with patients (LTLE and RTLE) performing significantly worse than healthy controls (*t* (42) = −4.16, *P* < 0.001), but no difference between both patient groups (*t* (42) = 1.17, *P* = 0.248). This corresponds very well to the results of the VLMT and the DCS. Especially, as fMRI behavioral data correlated significantly with verbal and nonverbal memory scores (VLMT: *r* = 0.458, *P* = 0.002; DCS: *r* = 0.515, *P* < 0.001). The reason for this lies most probably within the same area as the reason for the worse performance of both patient groups in the VLMT and the DCS. While it is known that RTLE patients suffer from nonverbal memory deficits, the reasons for the worse performance of LTLE patients than healthy controls have not yet been conclusively identified. The basis of this would be the underlying disease and the recurrent epileptic seizures which would lead to reorganization processes of some form. As the left mesial temporal lobe is involved in verbal memory, difficulties in verbalization, as described above, seem a plausible explanation.

### 3.2 fMRI whole brain results

**FIGURE 1:**
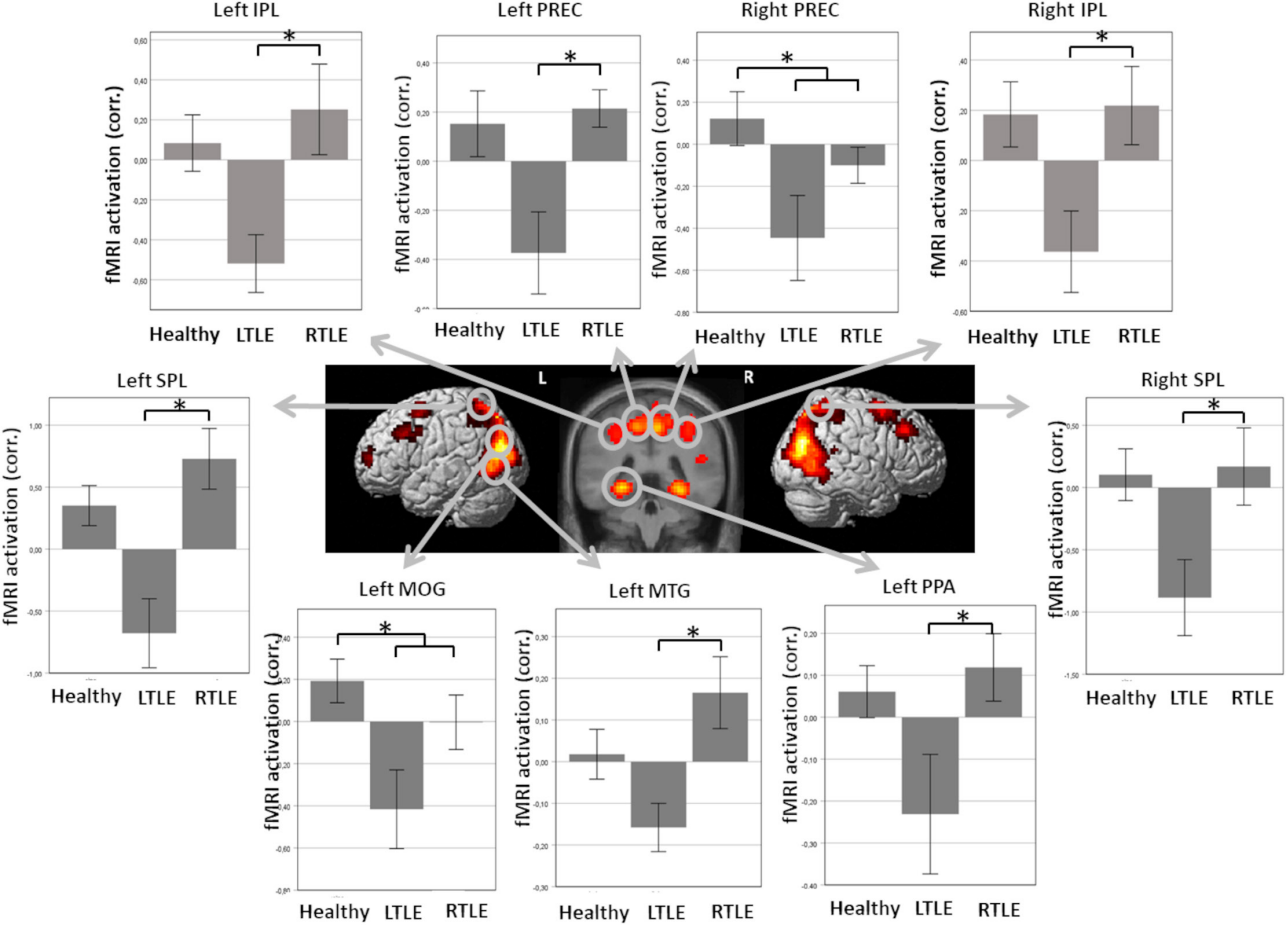
fMRI activations and comparisons between groups. fMRI activations within right and left SPL, right and left IPL, right and left PREC, right and left PPA, left MOG, and left MTG. Bar graphs demonstrate activations in all three groups. In the right and left SPL, LTLE patients activate significantly less than RTLE patients. In the right and left IPL, LTLE patients activate significantly less than RTLE patients. In the left PREC, LTLE patients activate significantly less than RTLE patients. In the right PREC, LTLE, and RTLE patients activate significantly less than healthy controls. In the left PPA, LTLE patients activate significantly less than RTLE patients. In the left MOG, LTLE, and RTLE patients activate significantly less than healthy controls. In the left MTG, LTLE patients activate significantly less than RTLE patients. *P < 0.05.

Second‐level analyses revealed in the group of healthy controls activations in a huge cluster comprising the bilateral middle and superior occipital gyrus, bilateral superior and inferior parietal lobulus, bilateral posterior middle temporal gyrus and bilateral precuneus, as well as the bilateral parahippocampal place area (PPA; gyrus parahippocampalis), bilateral frontal eye field (FEF; gyrus frontalis superior), and the bilateral middle frontal gyrus (Figure 1). Details of whole brain activations in healthy controls can be found in Table 3. In the group of LTLE patients, we observed activations in the right posterior middle temporal gyrus, right middle occipital gyrus, right precuneus, right superior occipital gyrus, right superior parietal lobulus, and left middle occipital gyrus. RTLE patients activated the posterior parts of the right inferior and middle temporal gyrus, right middle and superior occipital gyrus, right superior parietal lobulus, left middle occipital gyrus, and left precuneus (Figure 1). Based on these whole brain results, the following ROIs were defined for further analyses: middle occipital gyrus (MOG), superior occipital gyrus (SOG), superior parietal lobulus (SPL), inferior parietal lobulus (IPL), middle temporal gyrus (MTG), precuneus (PREC), parahippocampal gyrus (PHG/PPA), superior frontal gyrus (SFG/FEF), and middle frontal gyrus (MFG). FMRI activations for each ROI and group are shown in Table 4.

**TABLE 3:** Activated brain regions/clusters.

Brain regions	MNI coordinates	*Z* score	Cluster size
Healthy subjects (*N* = 28)			
Occipital Mid R/L, Occipital Sup R/L, Parietal Sup R/L, Parietal Inf R/L, Temporal Mid R/L, Precuneus R/L, ParaHippocampal R/L	39 −75 24	Inf	6183
Frontal Sup R (FEF)	24 5 54	6.04	417
Frontal Sup L (FEF)	−24 3 57	5.21	270
Frontal Mid R	42 36 36	4.76	169
Frontal Mid L	−33 60 9	4.62	57
Frontal Mid L	−51 27 36	4.58	168
LTLE (*N* = 12)			
Temporal Mid R, Occipital Mid R	51 −66 3	5.41	391
Precuneus R	15 −51 18	4.43	44
Precuneus R, Occipital Sup R, Parietal Sup R	21 −75 45	4.21	63
Occipital Mid L	−39 −78 21	3.73	145
RTLE (*N* = 9)			
Temporal Inf R, Temporal Mid R	36 −51 −9	4.58	61
Occipital Mid R, Temporal Mid R, Occipital Sup R	24 −78 −6	4.48	379
Occipital Mid L	−36 −84 9	4.45	374
Precuneus L	−9 −66 54	3.94	91
Parietal Sup R	18 −60 57	3.66	45

Abbreviations: Frontal Inf, inferior frontal gyrus; Frontal Sup, superior frontal gyrus; L, left; Occipital Mid, middle occipital gyrus; Occipital Sup, superior occipital gyrus; ParaHippocampal, gyrus parahippocampalis; Parietal Inf, inferior parietal lobulus; Parietal Sup, superior parietal lobulus; R, right; Temporal Mid, middle temporal gyrus.

**TABLE 4:** fMRI activations (beta estimates) in healthy subjects, LTLE and RTLE patients in each ROI.

ROI	Group mean (SEM)					
	Healthy subjects		LTLE		RTLE	
	Right	Left	Right	Left	Right	Left
MOG	1.46 (± 0.12)	1.20 (± 0.10)	1.00 (± 0.26)	0.57 (± 0.18)	1.24 (± 0.19)	0.95 (± 0.13)
SOG	1.22 (± 0.13)	0.87 (± 0.12)	0.67 (± 0.22)	0.34 (± 0.22)	0.87 (± 0.15)	0.65 (± 0.16)
SPL	1.91 (± 0.21)	1.55 (± 0.16)	0.67 (± 0.30)	0.26 (± 0.28)	1.25 (± 0.40)	1.20 (± 0.35)
IPL	0.27 (± 0.13)	0.37 (± 0.14)	−0.31 (± 0.16)	−0.26 (±0.15)	0.21 (± 0.17)	0.44 (± 0.24)
MTG	0.32 (± 0.07)	0.10 (± 0.06)	0.13 (± 0.07)	−0.10 (± 0.06)	0.25 (± 0.12)	0.16 (± 0.10)
PREC	1.45 (± 0.13)	1.22 (± 0.14)	0.63 (± 0.19)	0.44 (± 0.17)	0.50 (± 0.16)	0.56 (± 0.19)
PHG/PPA	0.15 (± 0.05)	0.15 (± 0.06)	0.15 (± 0.13)	−0.18 (± 0.15)	0.16 (± 0.10)	0.11 (± 0.08)
SFG/FEF	0.17 (± 0.07)	−0.03 (± 0.07)	−0.14 (± 0.08)	−0.14 (± 0.07)	0.04 (± 0.13)	0.11 (± 0.14)
MFG	0.30 (± 0.08)	0.25 (± 0.08)	−0.14 (± 0.17)	−0.01 (± 0.13)	0.10 (± 0.15)	0.33 (± 0.16)

Abbreviations: IPL, inferior parietal lobulus; LTLE, left temporal lobe epilepsy patients; MFG, middle frontal gyrus; MOG, middle occipital gyrus; MTG, middle temporal gyrus; PHG/PPA, parahippocampal gyrus/parahippocampal place area; PREC, precuneus; ROI, region of interest; RTLE, right temporal lobe epilepsy patients; SEM, standard error of the means; SFG/FEF, superior frontal gyrus/frontal eye field; SOG, superior occipital gyrus; SPL, superior parietal lobulus.

### 3.3 Comparison of activations between groups

Group comparisons using two‐sample *t*‐tests revealed increased activations in healthy controls compared to LTLE patients in the right cerebellum and the left inferior occipital gyrus (*P* < 0.001, corrected at cluster level, *k* > 80 voxels). LTLE patients on the other hand showed increased activations compared to healthy controls in the left and right precuneus (*P* < 0.001, corrected at cluster level, *k* > 102 voxels). Compared to RTLE patients, healthy controls show increased activations in the right precuneus (*P* < 0.001, corrected at cluster level, k > 261 voxels). All other comparisons did not reveal any suprathreshold activations.

Comparing activations within each ROI between groups using one‐way ANOVAs indicated that the three groups differed in the left MOG (*F*_(2, 46)_ = 5.15, *P* = 0.01, *ω* ^2^ = 0.15), right SPL (*F*_(2, 46)_ = 4.05, *P* = 0.024, *ω* ^2^ = 0.11), left SPL (*F*_(2, 46)_ = 8.30, *P* < 0.001, *ω* ^2^ = 0.23), right IPL (*F*_(2, 46)_ = 3.59, *P* = 0.035, *ω* ^2^ = 0.10), left IPL (*F*_(2, 46)_ = 4.21, *P* = 0.021, *ω* ^2^ = 0.12), left MTG (*F*_(2, 46)_ = 3.47, *P* = 0.039, *ω* ^2^ = 0.09), right PREC (*F*_(2, 46)_ = 3.42, *P* = 0.041, *ω* ^2^ = 0.09), left PREC (*F*_(2, 46)_ = 3.48, *P* = 0.039, *ω* ^2^ = 0.09) and left PPA (*F*_(2, 46)_ = 3.31, *P* = 0.045, *ω* ^2^ = 0.09) (Figure 1). Planned contrasts revealed that patients (LTLE and RTLE) activated significantly less in the left MOG (*t* (46) = −2.52, *P* = 0.015) and right PREC (*t* (46)= − 2.15, *P* = 0.036). LTLE patients activated significantly less than RTLE patients in the bilateral SPL (right: *t* (46) = −2.25, *P* = 0.029; left: *t* (46) = −3.70, *P* < 0.001), bilateral IPL (right: *t* (46) = −2.11, *P* = 0.040; left: *t* (46) = −2.55, *P* = 0.014), left MTG (*t* (46) = −2.60, *P* = 0.013), left PREC (*t* (46) = −2.15, *P* = 0.037), and left PHG/PPA (*t* (46) = −2.19, *P* = 0.034) (Figure 1). However, after adjusting the level of significance based on the Bonferroni correction, the three groups differed statistically significant only within the left SPL (*F*_(2, 46)_ = 8.30,  *P*< 0.001, *ω* ^2^ = 0.23) with LTLE patients activating significantly less than RTLE patients (*t* (46) = −3.70, *P* < 0.001). Activations in all other ROIs did not differ significantly between groups (each *P* > 0.05).

### 3.4 Correlation analyses

**FIGURE 2:**
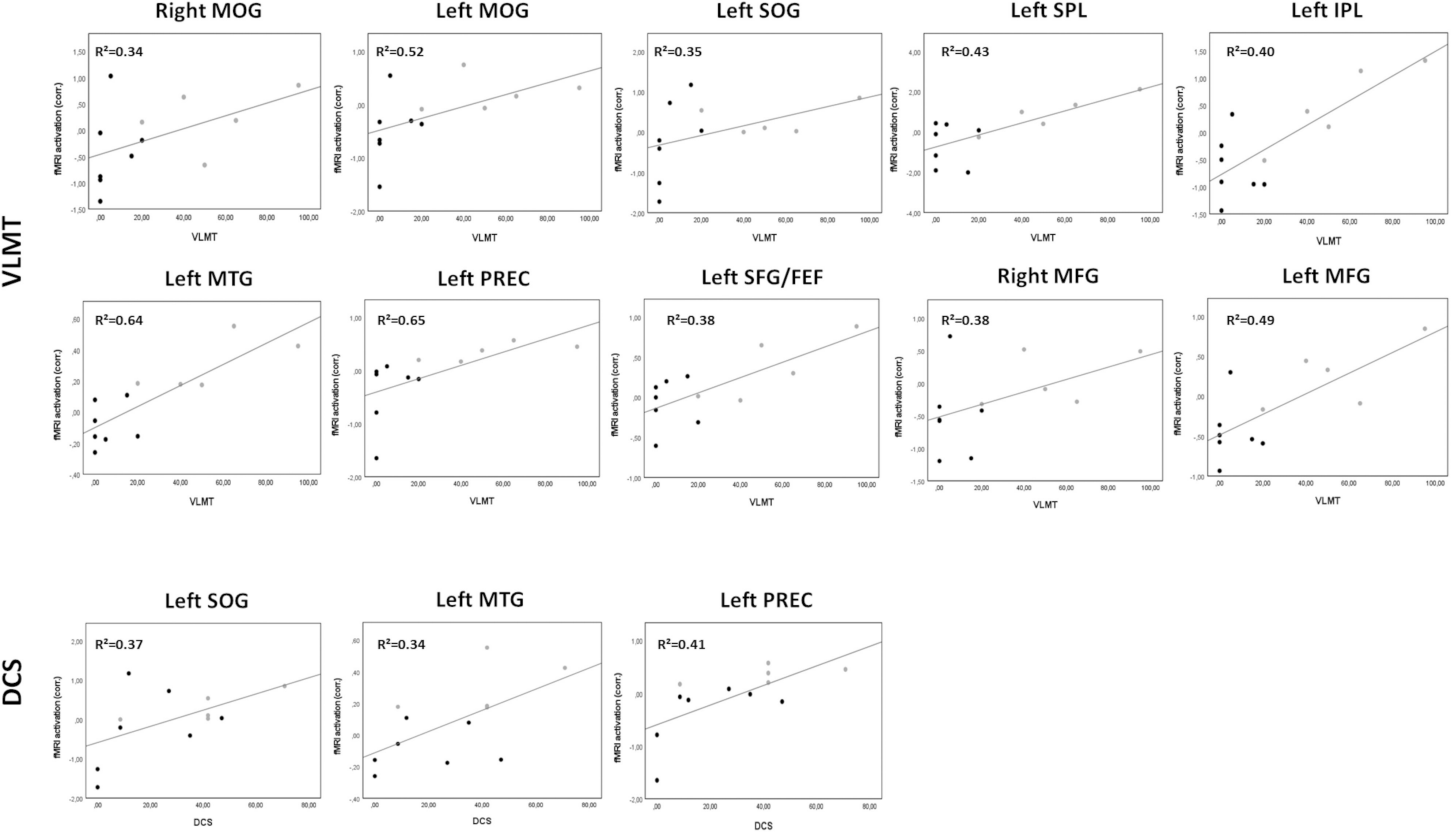
Correlations between activations and memory scores. Scatterplots demonstrate correlations between activations and memory scores, the black dots represent LTLE patients, and the gray dots represent RTLE patients. Verbal memory scores are linearly correlated with fMRI activations in the bilateral MOG, left SOG, left SPL, left IPL, left MTG, left PREC, left FEF, and bilateral MFG. Nonverbal memory scores show significant correlations with fMRI activations in the left SOG, left MTG, and left PREC.

Kolmogorov–Smirnov tests indicated that only activations within the left PREC were not normally distributed (*D* (12) = 0.282, *P* < 0.05), all other activations as well as the postoperative VLMT and DCS scores were normally distributed (*P* > 0.05). Correlation analyses between fMRI activations and postoperative memory scores across all groups (LTLE‐post and RTLE‐post) yielded linear correlations between VLMT scores and activations in the bilateral MOG (right: *r* = 0.581, *P* = 0.047; left: *r* = 0.720, *P* = 0.008). left SOG (*r* = 0.592, *P* = 0.043), left SPL (*r* = 0.653, *P* = 0.021), left IPL (*r* = 0.631, *P* = 0.028), left MTG (*r* = 0.802, *P* = 0.002), left PREC (*r* = 0.806, *P* = 0.002), left SFG/FEF (*r* = 0.620, *P* = 0.031), and bilateral MFG (right: *r* = 0.620, *P* = 0.031, *r* = 0.699, *P* = 0.011). The DCS score showed linear correlations with activations in the left SOG (*r* = 0.608, *P* = 0.036), left MTG (*r* = 0.587, *P* = 0.045), and left PREC (*r* = 0.643, *P* = 0.024). Activations in all other ROIs did not reveal any significant correlations with memory scores (each *P* > 0.05) (Figure 2). After correction for multiple comparisons, however, no correlations remained statistically significant.

Correlation analyses between age at epilepsy onset, duration of epilepsy, and seizure frequency and activations within each ROI yielded no statistically significant results, that is, our key activation effects are not mediated by any of these factors.

## 4 DISCUSSION

### 4.1 Role of the PMN in nonverbal memory encoding

Several meta‐analyses of encoding‐ and retrieval‐related effects have highlighted consistent recruitment of three distinct parietal regions supporting memory functioning: precuneus, mid‐cingulate cortex, and posterior inferior parietal lobule/dorsal angular gyrus.^3^, ^29^, ^30^, ^31^, ^32^ Activity in this so‐called parietal memory network, or PMN, has been shown to increase with repeated exposition to the stimuli and to be related to a higher memory performance while hippocampal activity and connectivity between hippocampus and PPC are strongest during initial encoding and decrease rapidly over time.^2^ These observations are in line with our results as we were able to demonstrate encoding‐related activity within the parietal cortex and the precuneus. Furthermore, activation within the left hemisphere ROIs correlated with postoperative verbal and nonverbal memory scores, supporting the hypothesis that stronger activity within the PMN is related to a higher memory performance but also indicating some sort of hemispheric lateralization within the PMN.

### 4.2 Parahippocampal place area

The PPA, firstly described by Epstein & Kanwisher,^33^ is a functional brain region that responds more strongly to images of scenes and places compared to other classes of visual stimuli and it is critical for scene and place recognition and navigation.^34^ Furthermore, the PHG is hypothesized to be important for spatial memory, including spatial layouts and the locations of objects in these environments.^35^ We observed reduced activity within the left PHG/PPA in LTLE patients, indicating that damage to the left HC leads to reduced activity within the ipsilateral PHG/PPA. Damage to the right HC does not seem to have a similar effect. So far, little is known about the effects of unilateral hippocampal damage on PHG/PPA function. In a study of patients with intractable epilepsy receiving small stereotaxic thermocoagulation lesions to either the left or right HC or the left or right PHG, the authors were able to show that damage to the right PHG causes impairments on an object‐location memory test, whereas damage to the right or left HC or the left PHG does not.^36^ In our study, we were not able to detect any behavioral differences regarding spatial memory functions between LTLE and RTLE patients. Therefore, the reduced activity within the left PHG/PPA in LTLE patients must probably be seen in the overall picture of reduced left hemisphere activations in this cohort.

### 4.3 Frontal eye field

The FEF is known to play a pivotal role in eye movements, especially saccadic eye movements for the purpose of visual field perception as well as the triggering of voluntary eye movements.^37^ We not only observed robust activations in the FEFs bilaterally but were also able to demonstrate that activity in the left FEF correlated significantly with postoperative verbal memory scores with stronger activations indicating better memory outcomes. Campana and colleagues^38^ demonstrated that FEF neurons are implicated in short‐term memory storage of spatial position. Wantz et al.^39^ used cTBS to interfere with the activity of the FEF just before a recall task and found that it impaired object recall. This study concurs with several studies supporting the so‐called “looking at nothing” phenomenon, which means that the retrieval of information from memory leads the gaze back to spatial locations that were previously associated with it.^40^ When eye movements are manipulated during memory retrieval, it has been shown that verbal memory retrieval is impaired.^41^ Our results are another piece of the puzzle linking FEF activity to memory function.

### 4.4 Differences in activation patterns between groups

Our healthy subjects showed rather symmetrical left and right hemisphere activations within the dorsal visual stream and the PMN, whereas LTLE patients activated significantly less within the left hemisphere. In RTLE patients, these alterations were less pronounced as they showed reduced activity only within the left MOG and the right PREC. As LTLE and RTLE patients did not differ significantly in memory scores or demographic factors, these differences in activation must be attributed to the hippocampal pathology, that is, left‐sided hippocampal sclerosis seems to have a clear effect on a left hemisphere network comprising the PMN as well as the PPA and the FEF. So far, little is known about lateralization within PMN and PREC. Furthermore, only a few studies have investigated the effects of TLE on visual encoding in extratemporal areas. Sidhu et al.^42^ observed in patients with left HS significantly fewer left hemisphere activations within the inferior frontal gyrus, lateral orbitofrontal cortex, MOG, amygdala, and hippocampus using a face encoding paradigm. Patients with right HS on the other hand showed significantly fewer right hemisphere activations within the middle frontal gyrus, MOG, and amygdala than control subjects. While the observations in the LTLE group are consistent with our findings, we did not observe a similar pattern in the RTLE group. This might be due to the smaller sample size in our study and the differences in the paradigm applied. Using a face‐name association task in a different study, we, too, observed reduced right hemisphere activity in RTLE patients alongside reduced activity within the left superior and inferior frontal gyrus in LTLE patients.^43^ Doll et al.^44^ investigated the functional correlates of verbal and nonverbal memory encoding and subsequent memory formation in a group of 31 LTLE and 25 RTLE patients compared to healthy controls. During scene encoding, they reported PMN activation across groups, but the activation did not predict subsequent memory. Furthermore, they observed reduced activation in the epileptogenic mesial temporal lobe in both patient groups and in the right lateral occipital lobe in RTLE patients. However, they also reported activation increases in extratemporal regions. Using an abstract pattern encoding task, Alessio and colleagues^45^ observed bilateral occipital and parietal activations in all three groups (healthy controls, LTLE, and RTLE), right frontal activations in healthy controls and bilateral frontal activations in the LTLE and RTLE group. However, their results were only reported qualitatively, that is, activation strength in each ROI was not compared between groups.

### 4.5 Correlation of activation with postoperative memory outcome

Activations within the bilateral MOG, left SOG, left SPL, left IPL, left MTG, left PREC, left SFG/FEF, and bilateral MFG correlated significantly with postoperative verbal memory scores. Nonverbal memory scores showed significant correlations with activations within the left SOG, left MTG, and left PREC. This indicates that the parietal cortex, including the precuneus, within the dominant hemisphere is not only qualitatively associated with memory encoding, but rather reflects memory functions quantitatively. Furthermore, the left SOG, left MTG, and left PREC seem to be convergence regions for the encoding of verbal and nonverbal material as they reflect verbal and nonverbal memory outcome equally. The bilateral MOG, left SPL, left IPL, left SFG/FEF, and the bilateral MFG on the other hand, seem to hold a significant material‐specific role in verbal episodic memory encoding.

One could even go one step further and use activation within these ROIs to predict postoperative memory outcome in TLE patients. However, to define activation thresholds for significant postoperative memory loss, a study with a larger patient cohort is needed. So far, only a few studies have examined the role of extratemporal regions in the prediction of postoperative memory outcome after mesial temporal resection. Using a verbal memory paradigm, Sidhu et al.^46^ observed that in LTLE patients, left frontal and anterior medial temporal activations correlated significantly with greater verbal memory decline postoperatively. Furthermore, it is known that activation in language‐relevant frontal areas correlates with verbal memory outcome after mTL resection.^47^ This coincides with our results as we were also able to demonstrate significant correlations between activations within the left frontal lobe and postoperative verbal memory outcome.

However, similar studies using nonverbal fMRI memory paradigms are lacking. While it has been shown that stronger activity within the PPC and the precuneus is related to a higher memory performance,^2^ our study is the first linking activation within the PMN to memory outcome after mTL resection.

### 4.6 Limitations

As HS is a rare condition, our sample size is rather small. This limited the interpretation of comparisons between groups on a whole brain level due to the ensuing multiple comparison problem. Therefore, we restricted our analyses to ROIs identified in the second‐level analyses. While this procedure clearly enhanced the sensitivity of our approach, it has the drawback of being blind for alterations outside the identified network.

Furthermore, compared to our patient cohort, the group of healthy controls is younger, larger and cognitively fitter. To account for the differences in age between groups, we removed variance correlated with participants' age from the extracted fMRI activations from each ROI in each participant by performing a simple regression analysis with age as independent variable and event‐related responses in each ROI as dependent variables and used only the regression residuals for further analyses. Regarding the behavioral data, we used the percentile ranks instead of the raw scores, that is, the standardized memory performance as compared to an age‐matched reference population, for the analyses. With regard to cognition, it is well known that patients with epilepsy show cognitive impairments compared to healthy controls. These impairments are very often already present at the time of epilepsy onset and increase with longer duration of epilepsy.^48^, ^49^, ^50^ This applies to both memory functions and IQ score.^48^ While a better match between the two cohorts would have been desirable, a certain discrepancy in this regard lies within the nature of the disease.

A somewhat surprising finding was that our patient cohorts showed no statistically significant differences between verbal and nonverbal memory scores, although the left hemisphere of the brain is generally more associated with language processing, whereas the right is associated with visuospatial and constructional tasks. A similar observation was made by Luelsberg et al.^51^ The reasons might lie in the rather small sample size of LTLE and RTLE patients and that it was assumed that right‐handed patients have a classical hemispheric functional organization, but no direct measure of hemispheric dominance was applied. Therefore, future investigations should aim for a higher number of patients and include measurements of patients' hemispheric lateralization with methods like language fMRI.

Although we were able to demonstrate clear correlations between activations and postoperative memory outcome, the sample size is also a major limiting factor here. Not only might it overestimate the relationship, it also limited the evaluation of distinct effects for left vs. right TLE.

### 4.7 Clinical relevance

Our study underlines the role of the PMN in spatial memory encoding. Our results demonstrate that LTLE patients with HS show significantly lower activations in areas belonging to the dorsal visual stream and the PMN, especially within the left hemisphere. We were able to show that activity in the left PREC, SOG, and MTG correlates with postoperative verbal and nonverbal memory scores. Activity in the left SPL, IPL and SFG/FEF as well as the bilateral MOG, and MFG correlates with postoperative verbal memory scores. We conclude that higher activations in areas belonging to the PMN and the dorsal visual stream, especially within the left hemisphere, before amygdalohippocampectomy may result in higher postoperative memory scores.

## FUNDING INFORMATION

This work was supported by the Fortüne‐Program (2055‐0‐1 and 2343‐0‐0) of the University of Tübingen.

## CONFLICT OF INTEREST STATEMENT

None of the authors has any conflicts of interest to disclose.

## ETHICAL APPROVAL

We confirm that we have read the Journal's position on issues involved in ethical publication and affirm that this report is consistent with those guidelines.

## PATIENT CONSENT STATEMENT

All participants gave written informed consent prior to participation in the study.

## Data Availability

All data from this study are available from the corresponding author upon reasonable request.
